# Berberine Chloride is an Alphavirus Inhibitor That Targets Nucleocapsid Assembly

**DOI:** 10.1128/mBio.01382-20

**Published:** 2020-06-30

**Authors:** Judy J. Wan, Rebecca S. Brown, Margaret Kielian

**Affiliations:** a Department of Cell Biology, Albert Einstein College of Medicine, Bronx, New York, New York, USA; Johns Hopkins Bloomberg School of Public Health

**Keywords:** berberine, RNA packaging, alphavirus, antiviral inhibitor, nucleocapsid assembly

## Abstract

Alphaviruses are enveloped positive-sense RNA viruses that can cause serious human illnesses such as polyarthritis and encephalitis. Despite their widespread distribution and medical importance, there are no licensed vaccines or antivirals to combat alphavirus infections. Berberine chloride (BBC) is a pan-alphavirus inhibitor that was previously identified in a replicon-based small-molecule screen. This work showed that BBC inhibits alphavirus replication but also suggested that BBC might have additional effects later in the viral life cycle. Here, we show that BBC has late effects that target the virus nucleocapsid (NC) core. Infected cells treated with BBC late in infection were unable to form stable cytoplasmic NCs or assembly intermediates, as assayed by gradient sedimentation. *In vitro* studies with recombinant capsid protein (Cp) and purified genomic RNA (gRNA) showed that BBC perturbs core-like particle formation and potentially traps the assembly process in intermediate states. Particles produced from BBC-treated cells were less infectious, despite efficient particle production and only minor decreases in genome packaging. In addition, BBC treatment of free virus particles strongly decreased alphavirus infectivity. In contrast, the infectivity of the negative-sense RNA virus vesicular stomatitis virus was resistant to BBC treatment of infected cells or free virus. Together, our data indicate that BBC alters alphavirus Cp-gRNA interactions and oligomerization and suggest that this may cause defects in NC assembly and in disassembly during subsequent virus entry. Thus, BBC may be considered a novel alphavirus NC assembly inhibitor.

## Abstract (precis)

The alphavirus chikungunya virus (CHIKV) is an example of an emerging human pathogen with increased and rapid global spread. Although an acute CHIKV infection is rarely fatal, many patients suffer from debilitating chronic arthralgia for years. Antivirals against chikungunya and other alphaviruses have been identified *in vitro*, but to date none have been shown to be efficacious and have been licensed for human use. Here, we investigated a small molecule, berberine chloride (BBC), and showed that it inhibited infectious virus production by several alphaviruses including CHIKV. BBC acted on a late step in the alphavirus exit pathway, namely the formation of the nucleocapsid containing the infectious viral RNA. Better understanding of nucleocapsid formation and its inhibition by BBC will provide important information on the mechanisms of infectious alphavirus production and may enable their future targeting in antiviral strategies.

## INTRODUCTION

Alphaviruses are enveloped, positive-sense, single-stranded RNA [(+)-ssRNA] viruses that belong to the family Togaviridae (reviewed in reference 1). The mature virion is ∼70 nm in diameter and contains an inner ∼40-nm diameter nucleocapsid (NC) core and an outer envelope glycoprotein layer, both arranged with *T*=4 icosahedral symmetry. The NC contains 240 copies of capsid protein (Cp) and a single ∼11.5-kb genomic RNA (gRNA). The core is surrounded by the viral envelope, a host-derived lipid bilayer containing 80 spikes composed of trimers of heterodimers of the E1 and E2 transmembrane proteins. The alphavirus genome contains a 5′ 7-methylguanosine cap and a 3′ polyadenylated tail, and it is divided into two open reading frames (ORFs). The first ORF is expressed from the 42S gRNA directly and encodes the 4 nonstructural proteins (nsP1 to nsP4). The second ORF is expressed from a 26S subgenomic RNA (sgRNA) that is colinear with the 3′ region of the genome and encodes 6 structural proteins (Cp, E3, E2, 6K, TF, and E1).

Alphaviruses infect cells through clathrin-mediated endocytosis and low pH-triggered membrane fusion, thus releasing the NC into the cytoplasm (reviewed in reference 2). The NC core is rapidly disassembled (3), and the gRNA is translated and processed to form the RNA replication complex (4). Replication occurs through a negative-sense RNA intermediate that serves as the template for gRNA and sgRNA production. The viral replicase complex is initially localized at the plasma membrane in membrane invaginations termed spherules, which can be internalized to form cytopathic vacuoles type I (CPVIs) in the cytoplasm (5).

The sgRNA is translated as a single polyprotein. Cp is translated first and immediately cleaved from the polyprotein in *cis* via its serine protease activity. Following release from the polyprotein, Cp is thought to associate with the 60S large ribosomal subunit in polysomes prior to its incorporation into assembling NCs (6, 7). The rest of the polyprotein is translocated across the endoplasmic reticulum membrane and processed by signal peptidase and furin in the secretory pathway. The E2 and E1 envelope proteins are delivered to the plasma membrane where virus budding occurs.

Cp is an ∼30- to 35-kDa protein with two domains joined by a conserved linker region (reviewed in reference 8). The Cp N-terminal domain is predominantly unstructured and contains a high concentration of basic residues that are thought to interact with the negatively-charged gRNA. A packaging signal present in the gRNA is proposed to facilitate its selective packaging into the viral NC (8, 9), producing virus particles with high specific infectivity. The C-terminal serine protease domain has a chymotrypsin-like fold and is organized as pentameric and hexameric capsomers on the surface of the NC (10, 11). A hydrophobic pocket on this domain specifically interacts with the endodomain of E2 in a 1:1 ratio during budding and in the mature virion (12, 13). Efficient virus assembly and budding from the plasma membrane require correct E2-E1, E2-Cp, and Cp-Cp interactions (reviewed in reference 14).

**FIG 1:**
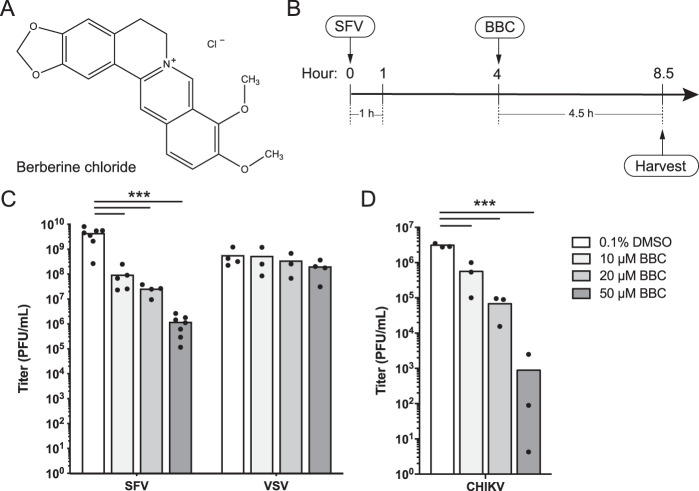
BBC inhibits production of infectious SFV and CHIKV. (A) Chemical structure of berberine chloride (BBC). (B) Timeline schematic of experimental design for treatment of SFV-infected cells. BHK-21 cells were infected with SFV at a multiplicity of infection (MOI) of 10 for 1 h, then treated with BBC at 4 h postinfection (hpi). Unless otherwise noted, all assays in this study were performed after 4.5 h of BBC treatment and at 8.5 hpi. (C) BHK-21 cells were infected with ppSFV or ppVSV at an MOI of 10, BBC was added at 4 hpi, and samples were harvested at 8.5 hpi. (D) BHK-21 cells were infected with CHIKV at an MOI of 1, BBC was added at 6 hpi, and samples were harvested at 12.5 hpi. (C and D) Infectious virus release was quantitated by plaque assay. The graphs represent the means with individual data points plotted for two to seven independent experiments. Two-way analysis of variance (ANOVA) (C) or one-way ANOVA (D) with Dunnett’s multiple-comparison test was performed to assess the statistical significance of infectious virus production in infected cells treated with vehicle control versus 10, 20, or 50 μM BBC. ***, *P* < 0.001.

Despite the medical importance of many alphaviruses, there are as yet no licensed human vaccines or antiviral treatments. Berberine chloride (BBC) (Fig. 1A) is a plant-derived isoquinoline alkaloid that has been used for centuries in Chinese and Ayurvedic medicine to treat dysentery and other illnesses (15). BBC was reported to inhibit diverse aspects of the lifecycles of (+)-ssRNA viruses of the Flaviviridae (16–18) and Picornaviridae (19–21) families. It has also been shown to bind various RNA and DNA structures *in vitro* (22–24).

BBC was previously identified in a screen of 3,000 small molecules using a cell-based CHIKV replicon reporter assay (25). BBC treatment reduces both viral RNA synthesis and protein expression in alphavirus-infected cells. Its exact mechanism of action is not yet well understood, as it had no effect on the replicase complex (25, 26). Rather, it may act through targeting host processes such as mitogen-activated protein kinase (MAPK) signaling, which is activated in CHIKV-infected cells (26). *In vivo* efficacy experiments show that BBC reduced viral load and decreased joint swelling in infected mice, suggesting that it could act as an antiviral agent (26).

Interestingly, time-of-addition studies showed that, in contrast to the other inhibitors identified in the replicon screen, BBC is still effective at reducing infectious particle production even when added relatively late in the alphavirus infection cycle (25). This result suggested that BBC might also have effects on postreplication steps such as genome packaging, particle assembly, or budding.

Here, we addressed the mechanism of BBC’s inhibitory effects on the late stages of alphavirus infection. Our cell culture and *in vitro* results demonstrate that BBC perturbs viral NC formation. Virions produced from BBC-treated cells have decreased infectivity despite control levels of particle production and genome packaging. BBC treatment also reduces the infectivity of free alphavirus particles. Together, our data support a model in which BBC targets Cp-gRNA interactions that are required for correct NC assembly and disassembly.

## RESULTS

### Effect of BBC on the production of infectious alphaviruses.

To investigate the late effects of BBC on the alphavirus life cycle, we infected baby hamster kidney 21 (BHK-21) cells with Semliki Forest virus (SFV) at a high multiplicity of infection (MOI) of 10 and added BBC at 4 h postinfection, when virus infection is robust but comparatively little particle release has occurred (27) (standard time course is shown in Fig. 1B). After a 4.5 h incubation with BBC, infectious particles in the culture medium were quantitated by plaque assay. BBC caused a dose-dependent inhibition of infectious SFV production (Fig. 1C) at drug and dimethyl sulfoxide (DMSO) vehicle concentrations that were not cytotoxic (see Fig. S1A and B in the supplemental material). Similar inhibition was observed in Vero cells (Fig. S1C) and in BHK-21 cells infected at different MOIs (data not shown). BBC treatment also significantly inhibited infectious CHIKV production (Fig. 1D). In contrast, BBC had no effect on production of infectious vesicular stomatitis virus (VSV), an enveloped negative-sense RNA virus that buds at the plasma membrane (Fig. 1C). Together, these data suggest that BBC targets a late step in the alphavirus life cycle or alters progeny virus infectivity.

10.1128/mBio.01382-20.1 FIG S1 BBC effects on uninfected cells and other infected cell lines. (A) Neutral red cytotoxicity test. BHK-21 cells were treated with the indicated concentrations of dimethyl sulfoxide (DMSO) (left) or BBC (right) for 8 h in medium A (black circles) or medium S (red triangles) or for 24 h in 2% fetal bovine serum (FBS) medium (green squares) or medium S (blue diamonds). Cells were then incubated with neutral red medium for 2 h, and dye uptake was quantified by absorbance at 540 nm. The values plotted represent the means for two independent experiments with three determinations per experiment, adjusted for background (no cells) and plotted as relative % values compared to nontreated cells. A dose-response inhibition curve was obtained, and 50% cytotoxic concentration (CC_50_) values were determined. hpt, h posttreatment. (B) BHK-21 cells were infected with ppSFV and treated with medium alone or with 0.1% DMSO, as in Fig. 1C. Infectious virus release was quantitated by plaque assay. The graph represents the means with individual points plotted for three independent experiments. A two-tailed Wilcoxon matched-pairs signed-rank test was performed to assess the statistical significance of infectious virus production of mock-treated infected cells versus vehicle-treated cells. *P* > 0.999, not significant. (C) Vero cells were infected with ppSFV at a multiplicity of infection (MOI) of 10 for 8.5 h. Infected cells were treated with BBC from 4 to 8.5 hpi. Infectious virus release was quantitated by plaque assay. The graph represents the means of two independent experiments with the individual data points shown. One-way analysis of variance (ANOVA) with Dunnett**’**s multiple comparison test was performed to assess infectious virus production in mock-treated infected cells versus vehicle control, 10, 20, or 50 μM BBC. **, *P* < 0.01. Download FIG S1, EPS file, 1.7 MB. Copyright © 2020 Wan et al. 2020 Wan et al. This content is distributed under the terms of the Creative Commons Attribution 4.0 International license.

### Effect of BBC on viral protein biogenesis.

**FIG 2:**
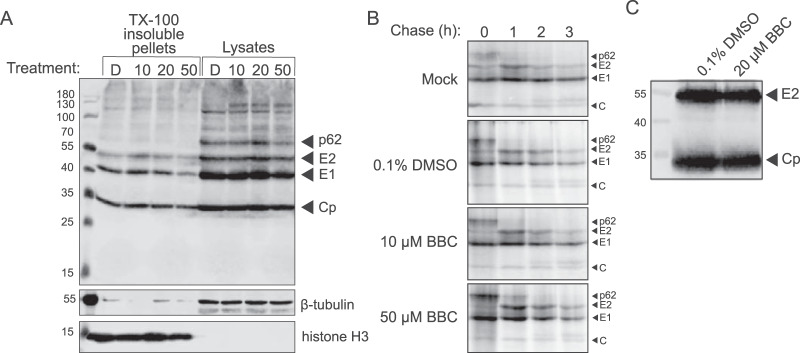
BBC does not affect structural protein synthesis or processing. (A) Steady-state structural protein production. BHK-21 cells were infected with ppSFV at an MOI of 10 and treated with dimethyl sulfoxide (DMSO) or BBC from 4 to 8.5 hpi. Cells were then solubilized in Triton X-100-containing lysis buffer and centrifuged to obtain detergent-insoluble pellets and lysates. Samples were analyzed by SDS-PAGE and Western blotting (WB) using polyclonal antibody (PAb) to E2 and E1 and monoclonal antibody (MAb) to Cp. Histone H3 and β-tubulin served as loading controls for the detergent-insoluble and cytoplasmic extracts, respectively. Treatment labels are as follows: D, 0.1% DMSO; 10, 10 μM BBC; 20, 20 μM BBC; 50, 50 μM BBC. (B) Pulse-chase analysis of protein synthesis and processing. BHK-21 cells were infected with ppSFV at an MOI of 10, treated with DMSO or BBC at 4 hpi, pulse labeled for 30 min with [^35^S]methionine/cysteine at 5 hpi, and chased for the indicated times, all in the continued presence of DMSO or BBC. At each time point, the cells were lysed, and the envelope proteins were immunoprecipitated with a PAb to E2 and E1. Samples were analyzed by SDS-PAGE and fluorography. (C) WB analysis of E2 to Cp ratio of pelleted particles. BHK-21 cells were infected with ppSFV at an MOI of 10 and treated with 0.1% DMSO or 20 μM BBC at 4 hpi, and the cell media were collected at 8.5 hpi. Particles were pelleted through a 20% sucrose cushion and resuspended in buffer. Samples were analyzed by SDS-PAGE and WB using MAb to E2 and PAb to Cp. The results shown in panels A, B, and C are representative of three independent experiments.

To confirm that the reduction in infectious alphavirus production was not due to impaired synthesis of sgRNA or viral structural proteins, we examined the steady-state structural protein levels in SFV-infected cells treated with DMSO or BBC as in Fig. 1B. Western blotting (WB) analysis showed comparable amounts of the structural proteins p62, E2, E1, and Cp in the cell lysates and the nuclear/detergent-insoluble pellets from BBC- versus DMSO-treated cells (Fig. 2A). A higher-molecular-weight band (∼130 kDa) was occasionally observed upon BBC treatment (Fig. 2A and data not shown), but its appearance was inconsistent, not dose dependent, and did not correlate with changes in the levels of Cp, E2, and E1. Pulse-chase analysis showed that the kinetics and extent of p62 processing to E2 were comparable in control and BBC-treated cells (Fig. 2B). WB analysis of pelleted particles produced from DMSO- or BBC-treated cells showed similar ratios of E2 to Cp (Fig. 2C), suggesting that BBC does not significantly alter virus protein composition. BBC treatment of cells infected with an SFV Δ6K/TF mutant (22, 28) showed inhibition similar to that of wild-type (WT) SFV (see Fig. S2 in the supplemental material), indicating that BBC does not act via effects on the expression of 6K or TF or their functional roles in budding. Immunofluorescence analysis of SFV-infected cells treated with DMSO or BBC showed similar E2/E1 localization at the plasma membrane, diffuse localization of Cp in the cytoplasm, and formation of intercellular extensions, as previously described (29) (see Fig. S3A and S3B in the supplemental material). Taken together, these results indicate that BBC treatment does not significantly impair structural protein production, localization, or proteolytic maturation.

10.1128/mBio.01382-20.2 FIG S2 BBC does not act via 6K/TF ribosomal frameshifting. BHK-21 cells were infected with Semliki Forest virus (SFV) wild type (WT) or SFV Δ6K/TF at an MOI of 10 and treated with 0.1% DMSO or the indicated concentration of BBC at 4 hpi, and infectious virus release at 8.5 hpi was quantitated by plaque assay. Results were plotted as reduction of titer compared to DMSO control, and represent the means of two independent experiments with the individual data points shown. Two-way repeated-measures ANOVA with Bonferroni’s multiple comparison test was performed to assess viral titer reduction in treated cells infected with SFV WT versus SFV Δ6K/TF. *P* > 0.2, not significant. Download FIG S2, EPS file, 0.8 MB. Copyright © 2020 Wan et al. 2020 Wan et al. This content is distributed under the terms of the Creative Commons Attribution 4.0 International license.

10.1128/mBio.01382-20.3 FIG S3 BBC does not affect localization of E2, E1, and Cp. Vero cells were infected with ppSFV at an MOI of 10 for 4 h, treated with 0.1% DMSO or 50 μM BBC, and fixed at 8.5 hpi. For cell surface staining (A), cells were not permeabilized before staining with MAbs to E2 and E1. In (B), cells were permeabilized and stained with antibodies to E2, E1, Cp, and tubulin. Nuclei were stained with Hoescht 33342 where marked. Cells were visualized by confocal microscopy. The images show merged Z-stacks with maximum intensity projection displayed and are representative of two independent experiments. Bars, 20 μm. Download FIG S3, PDF file, 0.4 MB. Copyright © 2020 Wan et al. 2020 Wan et al. This content is distributed under the terms of the Creative Commons Attribution 4.0 International license.

### Infectivity of virus particles released from BBC-treated cells.

**FIG 3:**
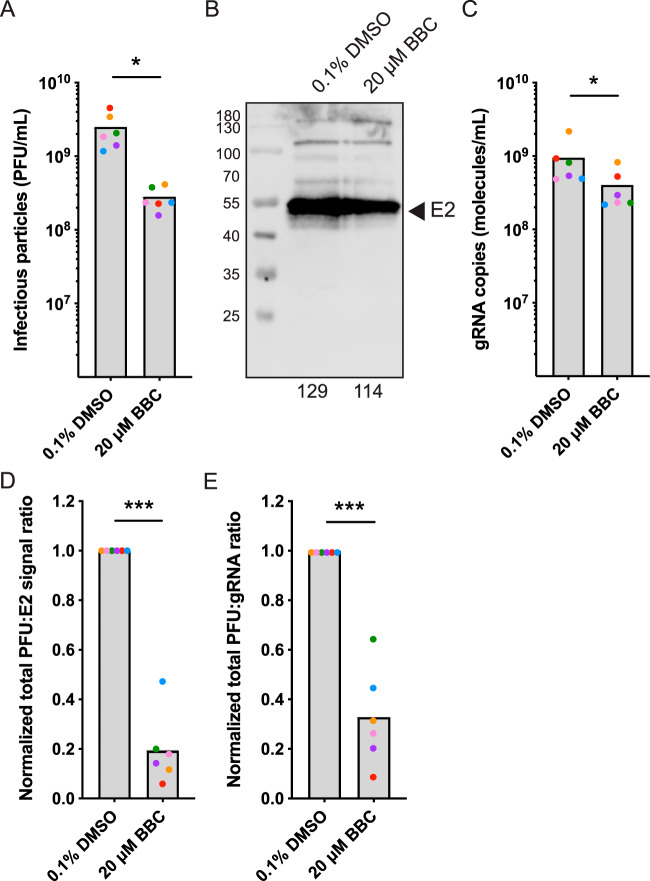
BBC treatment of infected cells decreases virus specific infectivity. BHK-21 cells were infected with SFV at an MOI of 10 and treated with 0.1% DMSO or 20 μM BBC at 4 hpi, and the cell media were collected at 8.5 hpi. (A) An aliquot of each sample was used to determine infectious particle number by plaque assay. The remaining sample was pelleted through a 20% sucrose cushion and resuspended in buffer. Parallel aliquots were analyzed for particle amounts by (B) SDS-PAGE and WB using a MAb to E2 or (C) used for RNA isolation and gRNA quantitation by RT-qPCR. For specific infectivity determination, the ratio of total infectious particle number to absolute E2 signal or to absolute gRNA copies (D and E, respectively) was expressed relative to DMSO treatment. The graphs represent the means with individual data points plotted for six independent experiments. Individual data points are paired for each treatment for each replicate, and paired points are displayed in the same color. The representative E2 WB and the quantitated signal displayed below in panel B is the sample corresponding to the orange point in panels A, C, D, and E. To assess the statistical significance between DMSO versus BBC treatment, two-tailed Wilcoxon matched-pairs signed rank tests were performed in panels A and C and two-tailed paired *t* tests were performed in panels D and E. ***, *P* < 0.001; *, *P* < 0.05.

We next investigated whether BBC’s inhibition of infectious virus production was due to a difference in particle production *per se* or to a decrease in particle specific infectivity. Infectious SFV released from BBC- or DMSO-treated cells was quantitated by plaque assay, and, in parallel, the amount of virus particles released was quantitated by both WB analysis of the particle E2 protein and reverse transcription quantitative real-time PCR (RT-qPCR) of the gRNA. SFV-infected cells treated with 20 μM BBC produced significantly less infectious virus (Fig. 3A; on average, 11% of control levels), but did not show a significant change in viral particles as determined by protein quantitation (Fig. 3B; on average, 80% of control levels). Particles showed a small but significant decrease in genome packaging (Fig. 3C; on average, 43% of control levels). Calculation of specific infectivity of the virus by comparing infectious units to either total particles (PFU:E2 signal) or genomes (PFU:genome) showed that particles produced from BBC-treated cells had significantly reduced specific infectivities that were on average 19% (PFU:E2 signal) and 33% (PFU:genome) of those of virus produced in control cells (Fig. 3D and E). Thus, our results indicate that while virus particle production was not significantly altered by BBC treatment, the virus produced in BBC-treated cells was less infectious than that from control cells.

### Effect of BBC on the morphology of virus infection.

We used transmission electron microscopy to examine the effect of BBC on the morphology of SFV-infected cells. Infected cells treated with DMSO (see Fig. S4A in the supplemental material) contained the characteristic replication-associated structures of spherules at the plasma membrane and cytoplasmic CPVIs. NCs were associated with the cytoplasmic vesicular structures known as CPVIIs and with the plasma membrane, where partial NCs were also observed. These observations support both NC-directed budding and E2/E1 glycoprotein-directed budding (30). BBC treatment did not detectably affect the replication complexes or the NCs that were associated with CPVII (Fig. S4B). However, while the electron microscopy evaluation was not quantitative, BBC appeared to reduce NCs in the cytoplasm while partial and complete NCs were observed budding at the plasma membrane. The morphology of released virus particles appeared similar when produced in control or BBC-treated cells.

10.1128/mBio.01382-20.4 FIG S4 Electron microscopy of control and BBC-treated infected cells. BHK-21 cells were infected with ppSFV at an MOI of 10, treated with (A) 0.1% DMSO or (B) 20 μM BBC from 4 to 8.5 hpi, and processed for electron microscopy. Examples of nucleocapsids in budding particles are denoted with closed black arrows, nucleocapsids associated with CPVIIs with closed white arrowheads, nucleocapsids at the plasma membrane with closed black arrowheads, nucleocapsids forming *de novo* at the plasma membrane with closed white arrows, spherules with open black arrowheads, and CPVIs with black asterisks. The results shown in panels A and B are representative of two independent experiments. Bars, 200 nm. Download FIG S4, PDF file, 0.5 MB. Copyright © 2020 Wan et al. 2020 Wan et al. This content is distributed under the terms of the Creative Commons Attribution 4.0 International license.

### Effect of BBC on nucleocapsid formation.

**FIG 4:**
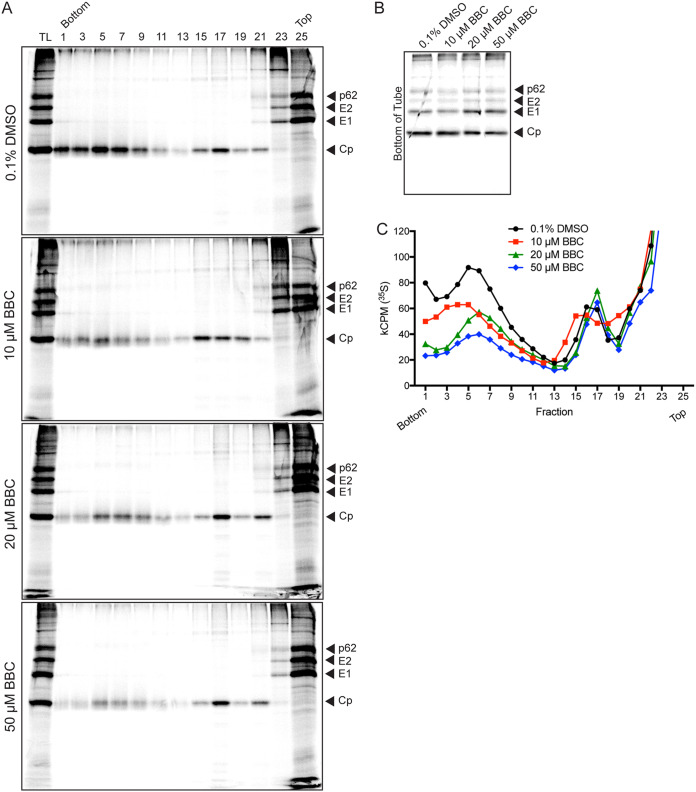
BBC treatment of infected cells decreases stable cytoplasmic nucleocapsids. BHK-21 cells were infected with ppSFV at an MOI of 10, treated with DMSO or BBC starting at 4 hpi, and then labeled with [^35^S]methionine/cysteine starting at 4.5 hpi in the continued presence of DMSO or BBC. At 8.5 hpi, cells were solubilized and analyzed by sucrose density gradient sedimentation. (A) Alternating fractions were analyzed by SDS-PAGE and fluorography, where TL represents equivalent aliquots of the total cell lysates. (B) The bottom of the tube was washed with SDS sample buffer to collect any Cp-bound complexes that pelleted through the 60% sucrose cushion, and analyzed as in panel A. (C) Radioactivity in all fractions was analyzed by scintillation counting. The Cp peak from fractions 3 to 9 represents cytoplasmic nucleocapsids, and the peak from fractions 15 to 19 represents Cp bound to ribosomes. The results shown in panels A, B, and C are representative of three independent experiments.

To more quantitatively address the effect of BBC on NC formation, we radiolabeled control or BBC-treated infected cells and analyzed the cell lysates by sucrose density gradient sedimentation. In agreement with prior studies by our group and others, Cp from control cells was found in fractions 3 to 9, which represent cytoplasmic NCs (31, 32), and in fractions 15 to 19, which represent Cp bound to the large ribosomal subunit (3, 6, 7) (Fig. 4A and C). Treatment of infected cells with BBC produced similar levels of total Cp (TL) and ribosome-associated Cp to those of DMSO-treated control cells, but a striking dose-dependent depletion of the cytoplasmic NC peak was observed. Cp depleted from the cytoplasmic NC fractions did not shift to another major fraction, but instead was most likely distributed across multiple fractions. Comparable amounts of Cp-containing aggregates were recovered from the bottom of the tube for all samples (Fig. 4B). Similar results were obtained if control and BBC lysates were analyzed on iodixanol gradients or if Cp was detected by WB analysis (data not shown). Thus, our data suggested that BBC altered stable NC formation, perhaps by acting on Cp-Cp or Cp-gRNA interactions or a possible host factor.

### Effect of BBC on virus-like particle production and nucleocapsid formation.

**FIG 5:**
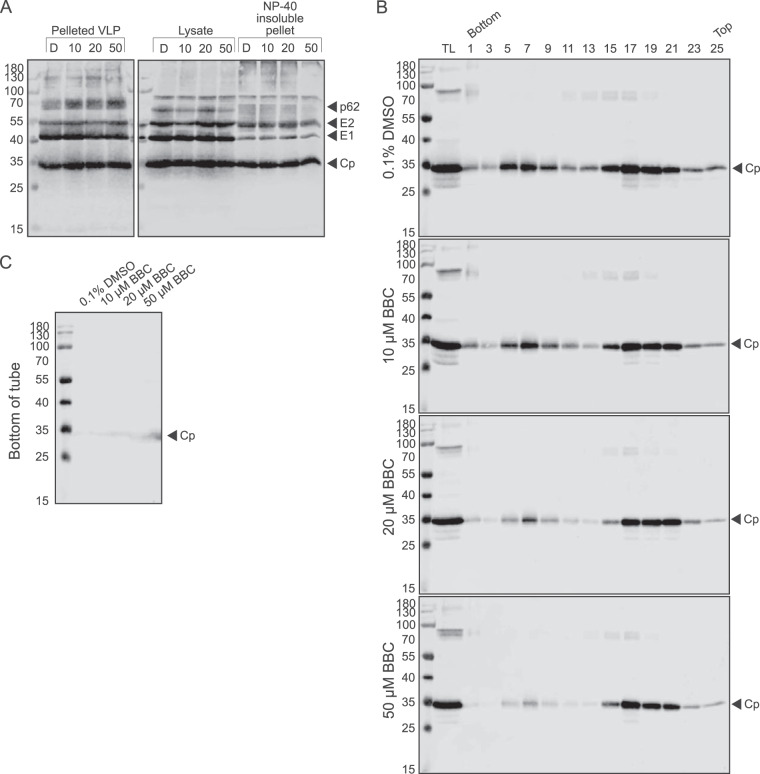
BBC treatment of VLP-producing cells decreases stable cytoplasmic nucleocapsids. (A) VLP and steady-state structural protein production. BHK-21 cells were transfected with a plasmid encoding the SFV structural proteins. At 22 h posttransfection, cells were washed and incubated with DMSO or BBC for 8 h. VLPs in the medium were pelleted through a 20% sucrose cushion, and cells were solubilized in NP-40-containing lysis buffer and centrifuged to obtain detergent-insoluble pellets and lysates. Samples were analyzed by SDS-PAGE and WB using PAb to E2 and E1 and MAb to Cp. Treatment labels are as follows: D, 0.1% DMSO; 10, 10 μM BBC; 20, 20 μM BBC; 50, 50 μM BBC. The results shown are representative of four independent experiments. (B and C) Gradient analysis of VLP cytoplasmic nucleocapsids. BHK-21 cells were treated as in panel A, and cell lysates were separated by iodixanol density gradient sedimentation. (B) Alternating fractions were analyzed by SDS-PAGE and WB analysis using PAb to Cp. TL represents equivalent aliquots of the total cell lysates; cytoplasmic nucleocapsids are in fractions 5 to 9, and fractions 17 to 21 represent Cp bound to ribosomes. (C) The bottom of the tube was washed with SDS sample buffer to collect any Cp-bound complexes that pelleted through the 50% iodixanol cushion and analyzed as in panel B. The results shown in panels B and C are representative of three independent experiments.

Expression of the alphavirus structural polyprotein in cells produces virus-like particles (VLPs) that bud from the plasma membrane and are structurally similar to authentic virus particles but which package cellular RNAs in their cores in place of the viral gRNA (33–35). We used the VLP system to test if the BBC effect was specific for NC containing the viral genome and/or produced in infected cells. As observed in virus-infected cells, VLP-expressing cells produced similar levels of viral proteins and assembled particles when treated with BBC or DMSO (Fig. 5A). We analyzed cytoplasmic NC by sedimentation on iodixanol gradients as both VLPs and their cytoplasmic NCs were less stable to sucrose gradient sedimentation. Similar to the results in virus-infected cells, expressing cells treated with BBC showed a dose-dependent decrease in cytoplasmic NCs, while the levels of ribosome-associated Cp remained similar to those in DMSO-treated cells (Fig. 5B and C). Together, our data support a model in which BBC affects the formation of cytoplasmic NCs.

### Effect of BBC on formation of 90S Cp intermediate.

**FIG 6:**
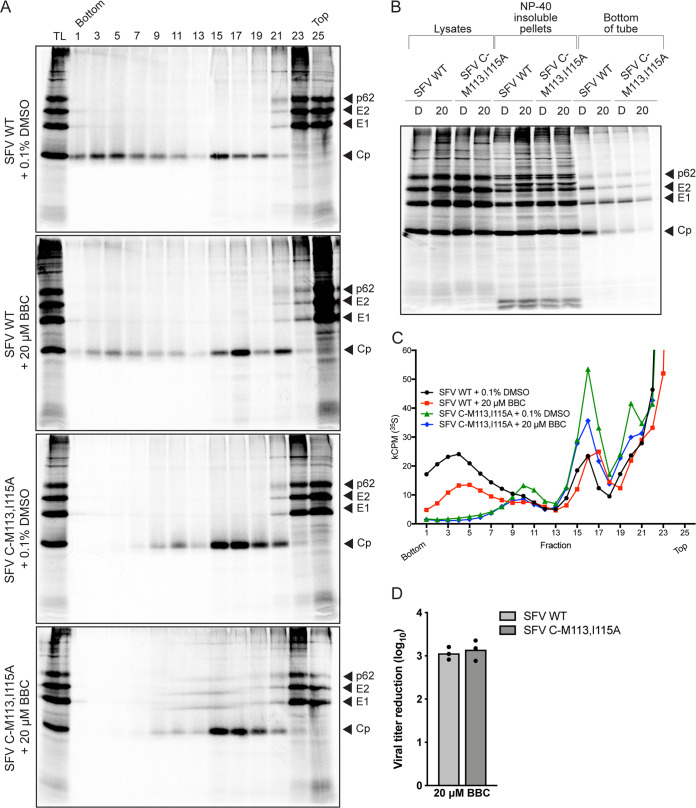
BBC treatment of SFV Cp mutant-infected cells decreases infectious virus production and the Cp 90S intermediate complex. (A, B, and C) Gradient analysis of viral cytoplasmic NC. BHK-21 cells were infected with SFV wild type (WT) or SFV C-M113,I115A mutant at an MOI of 10, incubated with 20 μM BBC as indicated, radiolabeled, and analyzed by density gradient sedimentation as in Fig. 4. (A) Alternating fractions were analyzed by SDS-PAGE and fluorography, where TL represents equivalent aliquots of the total cell lysates. (B) Structural proteins in cell lysates, detergent-insoluble pellets, and bottom of gradient tubes were analyzed by SDS-PAGE and fluorography. (C) Radioactivity in gradient fractions was analyzed by scintillation counting. The Cp peak at fractions 3 to 7 represents cytoplasmic nucleocapsids, the peak at fractions 10 and 11 represents the 90S intermediate complex, and the peak at fractions 15 to 19 represents Cp bound to ribosomes. Results shown in panels A to C are representative of two independent experiments. (D) Infectious virus production. BHK-21 cells were infected with SFV WT or SFV C-M113,I115A mutant at an MOI of 10 and treated with 0.1% DMSO or 20 μM BBC at 4 hpi, and the cell media were collected at 8.5 hpi. Infectious virus release was quantitated by plaque assay and plotted as reduction of titer compared to DMSO control. The graphs represent the means with individual data points plotted for three independent experiments. A two-tailed paired *t* test was performed to assess the statistical significance of viral titer reduction in treated cells infected with SFV WT versus SFV C-M113,I115A mutant. *P* = 0.281, not significant.

The ^113^MXI^115^ motif is located in the linker region of Cp and has been found to be important for Cp-Cp interactions (36, 37). The alanine substitution mutant SFV C-M113,I115A does not form stable cytoplasmic NCs in the cytoplasm but accumulates a 90S form that contains a high ratio of gRNA to Cp (37) and that may be an intermediate in NC assembly (32). We analyzed the effect of BBC on this intermediate in SFV C-M113,I115A mutant-infected cells. WT- or mutant-infected cells showed similar viral protein levels with or without BBC treatment (Fig. 6B). Gradient sedimentation showed that, as previously reported, mutant-infected cells lacked cytoplasmic NCs (fractions 3 to 7) but were enriched in the previously observed Cp-containing 90S complex (fractions 10 and 11; Fig. 6A and C) and also contained the ribosome-bound Cp pool (fractions 15 to 19). Treatment with 20 μM BBC decreased the 90S complex in mutant-infected cells, as well as the cytoplasmic NC peak in WT-infected cells. Plaque assays showed that BBC caused comparable reductions of WT and mutant infectious virus production (Fig. 6D). Together, these results suggest that BBC may target the formation of precursor NC complexes, thereby reducing the level of stable, fully-formed, cytoplasmic NCs.

### Effect of BBC on *in vitro* formation of gRNA core-like particles.

To determine whether BBC might be acting on potential host factors versus targeting Cp-Cp or Cp-RNA interactions, we evaluated the effect of BBC on the *in vitro* assembly of Cp into core-like particles (CLP) (38–40). CLP assembly is largely driven by electrostatics and charge neutralization of Cp’s N-terminal domain and can occur with negatively-charged cargo that includes ssRNA, single-stranded DNA (ssDNA) of ≥14 nucleotides, tRNA, and heparin (38, 39, 41–44). CLPs formed from purified recombinant Cp and nucleic acids are morphologically similar to isolated virus NCs (40, 42, 43).

Our assay was based on purified recombinant SFV Cp and *in vitro*-transcribed SFV gRNA (see Fig. S5 in the supplemental material). CLP formation was detected by dynamic light scattering (DLS), a relatively high-throughput method previously used to quantitate *in vitro* CLP assembly (42, 45), and by negative-stain electron microscopy. DLS measures the translational diffusion of molecules in solution due to Brownian motion (46). Fluctuations in light intensity over time yield an intensity autocorrelation function (ACF), from which the diffusion coefficient and hydrodynamic radius (*R_h_*) are calculated. Faster decay of the ACF indicates a smaller particle. Thus, changes in both *R_h_* and in the ACF are useful indicators to monitor CLP formation. Multimodal populations whose radii differ by at least 5-fold are resolved as separate peaks on a size distribution graph, while heterogeneity within peaks can be evaluated from the polydispersity (Pd) of the peak, where homogenous peaks exhibit a % Pd of <15, and heterogenous peaks show a % Pd of >30.

10.1128/mBio.01382-20.5 FIG S5 Validation of reagents for CLP assembly. (A) 2 μg of purified tobacco etch virus (TEV)-Strep, 2×Strep-Cp, and cleaved Cp analyzed by SDS-PAGE and stained with Coomassie brilliant blue R-250. L, prestained protein ladder. (B) Sucrose density gradient centrifugation of purified Cp alone, analyzed by SDS-PAGE and Western blotting (WB) analysis. L, prestained protein ladder; IN, input; BOT, bottom of tube. (C) Negative-stain electron micrograph of purified Cp (as in panel B). Note different magnifications. Bar, 100 nm. (D) *In vitro*-transcribed SFV gRNA analyzed on a native 1% (wt/vol) agarose Tris-acetate-EDTA (TAE) gel and visualized with ethidium bromide. 1, DNA ladder; 2, linearized infectious cDNA clone; 3, gRNA transcript after *in vitro* transcription; 4, gRNA after DNase I treatment; 5, gRNA after column purification. (E) *In vitro*-transcribed SFV gRNA after DNase I treatment and column purification, analyzed on a denaturing 1% (wt/vol) agarose MESA-formaldehyde gel and visualized with SYBR Gold. 1, RNA markers; 2, gRNA denatured with formamide and formaldehyde. (F) Folding of SFV gRNA after column purification of *in vitro*-transcribed RNA, analyzed on a native 1% (wt/vol) agarose TAE gel and visualized with ethidium bromide. 1, DNA ladder; 2, linearized infectious cDNA clone; 3, gRNA transcript after *in vitro* transcription; 4, gRNA after DNase I treatment; 5, gRNA after column purification; 6, gRNA after heat denaturation, renaturation, and refolding; 7, folded gRNA after freeze-thawing. Download FIG S5, PDF file, 2.9 MB. Copyright © 2020 Wan et al. 2020 Wan et al. This content is distributed under the terms of the Creative Commons Attribution 4.0 International license.

**FIG 7:**
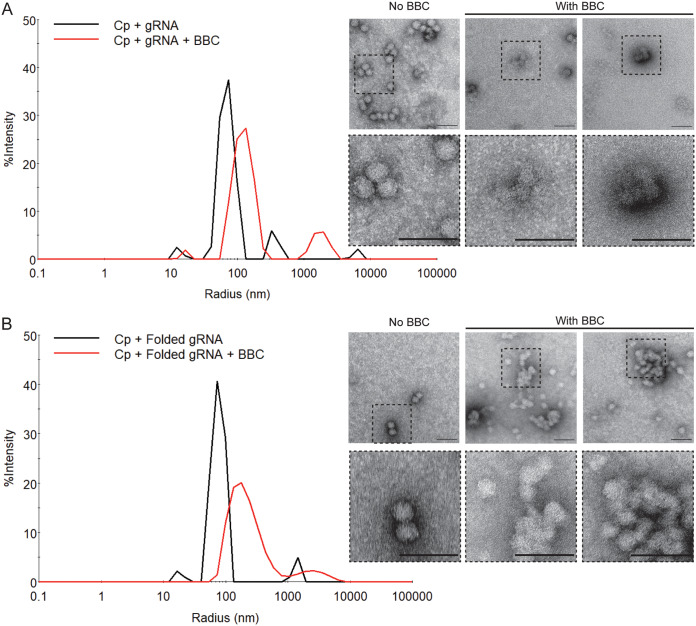
BBC inhibits *in vitro* formation of SFV gRNA CLPs. Recombinant SFV Cp was assembled *in vitro* with (A) SFV gRNA or (B) folded gRNA at a molar ratio of 720:1 Cp:RNA. This ratio represents a 3-fold Cp excess over that in the virus nucleocapsid and was chosen to ensure complete CLP formation. The gRNA was pretreated for 5 to 10 min with 250 μM BBC (representing a 100:1 BBC:Cp molar ratio). Cp was then added, and the extent of CLP assembly was analyzed. (Left) DLS spectra, with the *x* axis showing the hydrodynamic radius in nm. (Right) Negative-stain electron microscopy. Dashed boxes in the upper panels are shown at 3× higher magnification in the bottom panels. Bars, 100 nm. Results are representative of two independent experiments.

CLP assembly reactions were performed using both gRNA and folded, compacted gRNA to test for possible effects of BBC on RNA compaction. The gRNA preparation was column purified after *in vitro* transcription and was quasidenatured after elution, as evidenced by its slower migration pattern on an agarose gel (Fig. S5D, lane 4 versus lane 5). gRNA presumably contains an ensemble of RNA secondary structures, although its % Pd was 14.6 (see Fig. S6A in the supplemental material). We also prepared folded gRNA as described in Materials and Methods. Folded gRNA has been charge neutralized by Mg^2+^ ions, and its more compacted structure is demonstrated by its faster migration pattern on an agarose gel (Fig. S5F, lanes 6 and 7 versus lane 5) and its decreased *R_h_* (28 versus 36 nm) and greater homogeneity (% Pd = 12.1) in DLS analysis (Fig. S6A versus Fig. S6B). DLS analyses showed that both RNA preparations assembled with Cp to form CLPs, as shown by the rightward shift to a slower rate of decay in the ACF and an increase in *R_h_* (Fig. S6). Parallel negative-stain electron microscopy confirmed CLP formation and showed that the CLPs were ∼40 nm in diameter and appeared comparable for the two RNA preparations (Fig. 7, No BBC samples). Note that the CLP size differs when estimated by DLS versus negative stain, presumably reflecting differences in particle hydration and the inherent bias of DLS sensitivity toward larger particles. More heterogeneity was observed for CLPs formed with gRNA versus with folded gRNA, as the former showed a larger % Pd (57.8 versus 49.6%) and presented as a broader *R_h_* peak. However, the efficient CLP formation by both RNA preparations suggests that Cp binding was sufficient to compact gRNA that was not prefolded.

10.1128/mBio.01382-20.6 FIG S6 Validation of dynamic light scattering (DLS) for detection of CLP assembly. Recombinant SFV Cp was assembled *in vitro* with (A) SFV gRNA or (B) SFV folded gRNA at a 250:1 Cp:RNA molar ratio. (A and B, left) DLS spectra showing % intensity versus radius (nm), to compare the size distribution of the starting gRNA to that of the assembled CLP product. (Right) Corresponding autocorrelation functions of DLS spectra. The results shown are representative of four to six independent experiments. Download FIG S6, EPS file, 1.9 MB. Copyright © 2020 Wan et al. 2020 Wan et al. This content is distributed under the terms of the Creative Commons Attribution 4.0 International license.

The addition of BBC during CLP formation with either gRNA or folded gRNA resulted in a slower ACF decay rate (see Fig. S7A and B in the supplemental material) and shift to higher *R_h_* (Fig. 7). Negative-stain analysis of the BBC-treated gRNA assembly reactions detected fewer CLPs overall and visualized some normal-appearing CLPs and structures that included crescent-shaped incomplete particles (Fig. 7A). In contrast, BBC treatment during the assembly of folded gRNA into CLPs resulted in large aggregates, which could represent nearly complete CLPs that are a later step in the assembly process (Fig. 7B). Thus, our results suggest that BBC treatment during *in vitro* assembly of SFV Cp with gRNA impairs the formation of fully formed CLPs and potentially traps the process in intermediate stages of assembly. Most importantly, our *in vitro* results support a model for a viral component rather than a cellular factor as BBC’s target.

10.1128/mBio.01382-20.7 FIG S7 Autocorrelation function of CLP assembly with SFV gRNA. Autocorrelation functions for DLS spectra of CLPs assembled from (A) SFV gRNA and (B) folded gRNA with and without BBC (from Fig. 7). Download FIG S7, EPS file, 1.3 MB. Copyright © 2020 Wan et al. 2020 Wan et al. This content is distributed under the terms of the Creative Commons Attribution 4.0 International license.

### Effect of BBC on *in vitro* formation of DNA-containing core-like particles.

Unlike the specific gRNA packaging in infected cells, *in vitro* CLP assembly can occur efficiently with different lengths, sequences, and types of nucleic acids (38, 39, 43). We tested the effects of BBC on Cp assembly with a well-studied nucleic acid cargo, a 48-nucleotide ssDNA oligomer (39, 40, 43, 44, 47). This 48-mer has a predicted hairpin motif that is hypothesized to serve as a scaffold that bridges two Cp monomers and neutralizes the basic residues in Cp’s N terminus (43). It is unclear how such 2:1 Cp:DNA complexes then assemble into a complete CLP. Cp and the 48-mer were incubated together at a 2:1 Cp:DNA molar ratio, and the extent of CLP assembly was evaluated by DLS and negative-stain electron microscopy. The 48-mer ssDNA was efficiently assembled into CLPs, while preincubation of the 48-mer with BBC produced a dose-dependent alteration in CLP formation (see Fig. S8 in the supplemental material). Negative-stain electron microscopy of the BBC-treated samples showed mostly complexes with incorrect geometry and curvature, consistent with the appearance of distinct multimodal peaks and a larger *R_h_* in DLS measurements (Fig. S8). Thus, our results indicate that BBC can also perturb the CLP assembly of noncognate nucleic acids such as the 48-mer, in agreement with BBC’s inhibition of stable cytoplasmic NC formation in VLP-expressing cells.

10.1128/mBio.01382-20.8 FIG S8 BBC inhibits the formation of CLPs with single-stranded DNA (ssDNA) 48-mer. Recombinant SFV Cp was assembled *in vitro* with a ssDNA 48-mer at a 2:1 Cp:DNA molar ratio in the presence of BBC, as described for Fig. 7. The extent of assembly was then analyzed by DLS and visualized by negative-stain electron microscopy. (A, left) DLS spectra showing % intensity versus radius (nm) to compare the size distribution of CLPs assembled from a 48-mer with and without the indicated BBC molar ratios. (Right) Corresponding autocorrelation functions of DLS spectra. (B) Negative-stain electron micrograph of assembly reactions. The with BBC sample shows the 2 Cp:1 48-mer:150 BBC (representing 187.5 μM BBC) sample. Dashed boxes in the upper panels are shown at 3× higher magnification in the bottom panels. Bars, 100 nm. The results shown in panels A and B are representative of two independent experiments. Download FIG S8, PDF file, 2.5 MB. Copyright © 2020 Wan et al. 2020 Wan et al. This content is distributed under the terms of the Creative Commons Attribution 4.0 International license.

To evaluate whether BBC inhibits the initial interaction of Cp and nucleic acid or the assembly of Cp-nucleic acid complexes into CLPs, we tested the effect of BBC in a competitive enzyme-linked immunosorbent assay (ELISA) (see Fig. S9 in the supplemental material). The 48-mer oligo with a 3′ biotin tag was preincubated with serially diluted concentrations of BBC starting at a maximal molar ratio of 1:400. The 48-mer-BBC mixtures were then incubated for 1 h at 37°C with plate-immobilized Cp at a 10-fold molar ratio of 48-mer:Cp. The immobilized Cp is unable to oligomerize or assemble but is free to bind the 48-mer, and binding can then be detected by addition of horseradish peroxidase (HRP)-conjugated streptavidin. The binding of 48-mer to Cp was unchanged by the presence of increasing concentrations of BBC (Fig. S9B). These results suggest that BBC does not act on initial Cp-nucleic acid interaction but rather prevents the correct oligomeric assembly of Cp-nucleic acid complexes.

10.1128/mBio.01382-20.9 FIG S9 BBC does not inhibit Cp-nucleic acid interaction. (A) Validation of enzyme-linked immunosorbent assay (ELISA). Wells were incubated with the indicated mixtures for 1 h, washed and blocked with 1% BSA, and reacted with streptavidin-horseradish peroxidase (HRP) (except for the Cp [TMB only] and empty well reading [none] samples). The reactions were stopped with sulfuric acid after 15 min to give maximal sensitivity for background detection, and absorbance was measured at 450 nm. Background signals of Cp plus 48-mer and Cp alone were evaluated compared to Cp plus 48-mer–3′ biotin binding. Plate chemistry was evaluated for any nonspecific binding of 48-mer–3′ biotin, 48-mer, and streptavidin-HRP compared to empty well readings (none). Cp (TMB only) serves as a negative control for any potential background absorbance of TMB substrate. The graph represents the mean absorbance readings with individual points plotted for two independent experiments. (B) Competitive ELISA. The 48-mer–3′ biotin was preincubated with the indicated BBC concentrations for 5 to 10 min at room temperature. The mixture was then incubated for 1 h at 37°C with wells that were precoated with Cp. Plates were washed and blocked with 1% BSA, and bound biotin was detected with HRP-coupled streptavidin. The reaction was stopped with sulfuric acid after 15 min and absorbance measured at 450 nm. The values were adjusted for nonspecific background signals using mean values determined for Cp plus unlabeled 48-mer, and then the ratio of the absorbance was plotted relative to that with no treatment. A dose-response with a least-squares fit was applied using a nonlinear regression model. The points represent the mean absorbance readings with standard deviation error bars for two independent experiments with three repetitions per experiment. Download FIG S9, EPS file, 1.0 MB. Copyright © 2020 Wan et al. 2020 Wan et al. This content is distributed under the terms of the Creative Commons Attribution 4.0 International license.

### Direct effect of BBC treatment on virion infectivity.

**FIG 8:**
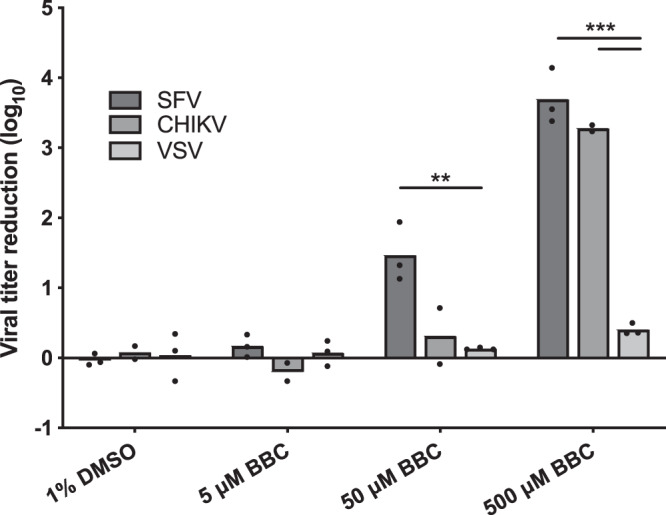
Direct BBC treatment inhibits the infectivity of SFV and CHIKV virions. Stocks of ppSFV, CHIKV, and ppVSV were treated with the indicated DMSO or BBC concentrations for 1 h at 37°C. Infectious virus titer was quantitated by 50% tissue culture infective dose (TCID_50_) assay, and viral titer reduction was calculated based on the log difference between control and treated samples. The graphs represent the means with individual data points plotted for two to three independent experiments. Multiple *t* tests were performed to assess the statistical significance of viral titer reduction between viruses under each treatment condition. ***, *P* < 0.001; **, *P* < 0.01.

To investigate if BBC could also target Cp-Cp and/or Cp-gRNA interactions in virus particles, we tested for direct effects of *in vitro* BBC treatment on alphavirus infectivity. High-titer virus stocks of SFV, CHIKV, and VSV were incubated with increasing concentrations of BBC for 1 h at 37°C. The infectious titer was then determined by 50% tissue culture infective dose (TCID_50_) assay after dilution of residual BBC down to the nM or pM range, concentrations that were shown not to inhibit virus entry or replication (25, 26). While treatment with 5 μM BBC had no effect, concentrations of 50 or 500 μM caused significant reductions in SFV and CHIKV infectivity without decreasing VSV infectivity (Fig. 8). Thus, external BBC treatment reduces alphavirus infectivity, potentially by targeting already established Cp-Cp and Cp-gRNA interactions in the free virus particle.

## DISCUSSION

Our results show that BBC treatment relatively late in the virus life cycle significantly inhibited the production of infectious SFV and CHIKV but not that of VSV. While most aspects of alphavirus infection, including protein and particle production, were unchanged, gradient sedimentation analyses showed that BBC decreased stable NC levels in both virus-infected and VLP-expressing cells. BBC treatment of SFV C-M113,I115A mutant-infected cells decreased production of the 90S complex, a putative NC nucleation intermediate. BBC also inhibited the formation of CLP *in vitro*, arguing against BBC’s targeting of a host cell factor. Together, our data support a model in which BBC inhibits the concerted assembly of Cp-nucleic acid complexes.

Specific infectivity determinations showed that BBC treatment of alphavirus-infected cells caused only small differences in gRNA packaging but strongly reduced progeny virus infectivity. A potential explanation is that BBC is incorporated into the NC, leading to improper Cp-gRNA interactions, oligomerization, and NC misassembly in infected cells. Disordered and irregular NCs with imperfect icosahedral symmetry have been observed in flaviviruses and alphaviruses (42, 48–51) and have been postulated to affect the uncoating and disassembly of the incoming viral NC. By altering NC assembly, BBC may affect the architecture, stability, or function of the incoming gRNA. Cytoplasmic NCs have been proposed to undergo a maturation step upon envelopment into virus particles (52–54). We found that *in vitro* BBC treatment of alphavirus particles dramatically reduced virus infectivity, while VSV was highly resistant. The BBC sensitivity of alphavirus particles argues that their Cp-gRNA interactions and NC conformation are also vulnerable to BBC disruption. Thus, BBC may perturb Cp-nucleic acid interactions that occur throughout the alphavirus life cycle, providing a rationale for BBC’s impairment of both stable NC formation and virus viability and suggesting that shared mechanisms act during both assembly and disassembly.

### *In vitro* intermediates.

Small-molecule inhibitors could theoretically target any step of NC assembly to alter the overall outcome. Recently, a two-stage assembly model has been proposed for NC assembly of (+)-ssRNA viruses (55, 56). The model posits genome condensation by specific Cp-gRNA and Cp-Cp interactions, followed by cooperative nucleation, a slower process in which additional Cp molecules are assembled on the gRNA through Cp-Cp interactions and lower-affinity Cp-gRNA interactions. We compared the effects of BBC on CLP assembly using SFV gRNA versus folded SFV gRNA, which should bypass the genome condensation step. BBC blocked CLP formation with either RNA, leading to what we propose are either locked intermediate stages of CLP assembly or off-pathway intermediates, such as kinetic traps, that lead to misassembled CLPs. Since CLP assembly with both SFV gRNA species was sensitive to BBC treatment, this suggests that BBC affects a postcondensation step. Furthermore, the trapped intermediates appeared to have the correct curvature, suggesting that BBC targets a later step in the assembly process. The short length of the 48-mer ssDNA enables rapid Cp binding through electrostatic interactions, thus bypassing genome condensation and presumably driving assembly via Cp-Cp interactions. 48-mer CLPs were observed to be nucleated in the presence of BBC, but with incorrect curvature. This suggests that BBC affects later stages of assembly, either by inhibiting nucleated oligomers from assembling into CLP or by pushing assembly pathway trajectories toward kinetic traps. Such models could also explain BBC’s ability to alter stable NC formation in VLP-expressing cells, which presumably contain RNAs of considerably smaller size and lower affinity. While much remains to be determined about the process of alphavirus NC assembly, our results with BBC indicate that inhibitors may be useful tools to trap distinct intermediates in NC assembly both *in vitro* and in infected cells.

### The multiple effects of BBC on alphavirus infection.

Careful studies by Varghese et al. previously showed that BBC inhibits early steps in alphavirus replication and suggested that it might have additional effects later in the infection life cycle (25). We found that BBC affected NC assembly in cell culture and *in vitro* and that it decreased virus infectivity via treatment of either infected cells or free viral particles. We propose that BBC targets important Cp-gRNA oligomerization and rearrangement steps. It is not clear if the early effects observed by Varghese et al. could be related to BBC’s actions on such Cp-gRNA interactions. Our assays followed the late effects of BBC after infection at a high MOI and treatment with 20 μM BBC from 4 h postinfection. The early effects on replication were observed when cells were treated with 3 to 20 μM BBC concomitantly with virus infection at either a low or a high MOI (25, 26). Interestingly, while treatment with BBC early in infection reduces gRNA and sgRNA synthesis and viral protein levels in infected cells, no decrease is observed in a *trans*-replication system (25, 26). This system is based on separate plasmids, one encoding the nonstructural proteins and one encoding a reporter RNA template. The *trans*-replication results indicated that replicase activity *per se* is not directly affected by BBC treatment, and thus it was proposed that BBC may act by targeting a host factor that is involved in replication during virus infection but not in the *trans*-replication system. However, an alternative explanation could be that reporter RNA templates in alphavirus *trans*-replication systems are constructed to contain the specific sequence elements required for replication (57) and thus differ substantially from the more complex full viral genome (58, 59). If BBC targets sequences or structures found only in the gRNA, the observed difference between viral RNA replication versus *trans*-replication could reflect BBC’s interactions with the gRNA. It is thus possible that BBC’s early inhibition of viral RNA replication is related to the late effects we observe on Cp-RNA interactions.

Nevertheless, our data together with that of the Ahola group suggest that BBC has multiple effects on alphavirus infection. In addition to its effects on RNA replication and NC assembly, BBC also attenuates the alphavirus-induced host MAPK signaling pathway (26). The Ahola group isolated an SFV mutant that contains two mutations in nsP4, the RNA-dependent RNA polymerase, and that is partially resistant to early inhibition by 3 μM BBC (60). However, we found that the nsP4 mutant was not resistant to late, higher-dose BBC treatment, and we were unable to isolate such late-effect SFV mutants (data not shown). The late effects we describe are likely to be more complex than simple targeting of Cp-gRNA interactions, given that even when gRNA is not present, BBC nonetheless affects NC formation in VLP-expressing cells and *in vitro* assembly of the 48-mer CLP. Thus, BBC exhibits a novel mechanism of action that has not been previously described and could provide a new targeting strategy for therapeutics.

### Development of antiviral strategies.

Antivirals that target the late steps of alphavirus infection have focused on inhibitors that bind the hydrophobic pocket of Cp’s protease domain, preventing its interaction with the endodomain of E2. Structure-based molecular docking studies have identified several single heterocyclic ring compounds and polycyclic compounds that contain flexible hydrocarbon linkers between single aromatic rings as potential antivirals that target Cp’s hydrophobic pocket with high affinity (61–66). However, bulky polycyclic compounds with a complex planar ring system, such as BBC, have not been shown to dock in the pocket. Indeed, our data here show that BBC does not interfere with E2-Cp interactions during budding; they instead support a mechanism of action in which BBC targets Cp-Cp or Cp-gRNA interactions during NC assembly and disassembly.

Potent antiviral inhibitors with bimodal effects in the early and late stages of infection have been described for other viral systems. For example, PF-1385801, a small-molecule inhibitor discovered in an HIV-1 replication screen, prevents viral uncoating, reduces particle infectivity, and alters nascent virion morphology by increasing the rate of Cp multimerization (67). ST-148, a small-molecule inhibitor with broad-spectrum activity against all dengue virus serotypes, inhibits NC disassembly and reduces particle production through stabilization of Cp-Cp interactions (68, 69).

Our work here suggests that, late in alphavirus infection, BBC acts to inhibit the concerted assembly of Cp-nucleic acid oligomers. Drugs that target the Cp of (+)-ssRNA viruses have been found to be relatively unlikely to select for virus resistance. Although drug-resistant Cp variants arise within infected cells, they form mixed oligomers with drug-sensitive Cp. Such mixed assemblies remain drug-sensitive, preventing the drug-resistant variant from outcompeting the drug-susceptible genome (70, 71). Inhibitors that bind the gRNA and interfere with its packaging are also predicted to be less likely to select for viral resistance (72). Given these points, the lack of isolation of a BBC-resistant mutant under our late selection conditions is perhaps not surprising. Since the generation of viral drug resistance has been a major issue in the development of effective antivirals, BBC and compounds with similar activities may be attractive candidates for future development.

## MATERIALS AND METHODS

### Infectious clones, mammalian expression vectors for viral proteins, and recombinant protein expression plasmids.

The SFV infectious clones pSP6-SFV4 and pSP6-SFV4Δ6K/TF were provided by Peter Liljeström and Henrik Garoff (Karolinska Institute) (73). The SFV mutant C-M113,115A was generated from pSP6-SFV4 by overlap extension PCR. All CHIKV studies used virus derived from the CHIKV vaccine strain 181/25 infectious clone pSinRep5-181/25ic, provided by Terence Dermody (University of Pittsburgh) (74). The SFV structural protein expression plasmids pcDNA3.1-Cp626KE1 and pcDNA3.1-p626KE1 were previously described (29). The pET-29a(+) vector was provided by Chris Lima (Memorial Sloan Kettering Cancer Center). The SFV Cp expression construct was generated by overlap extension PCR and cloned into pET-29a(+) after an N-terminal double Strep tag, a glycine-serine linker, and a tobacco etch virus (TEV) protease cleavage site (2×Strep-Cp). A pET-23d(+) expression construct for TEV protease with a C-terminal Strep tag (TEV-Strep) was provided by Félix Rey (Institut Pasteur).

### Cells.

Baby hamster kidney 21 (BHK-21) cells (75) were cultured in Dulbecco’s modified Eagle’s medium (DMEM) containing 25 mM d-glucose, 2 mM l-glutamine, 10% tryptose phosphate broth, 5% fetal bovine serum (FBS), 100 U/ml penicillin, and 100 μg/ml streptomycin. Vero cells were cultured in DMEM containing 25 mM d-glucose, 4 mM l-glutamine, 1 mM sodium pyruvate, 10% FBS, 100 U/ml penicillin, and 100 μg/ml streptomycin. All cells were cultured at 37°C with 5% CO_2_ and confirmed to be free of mycoplasma contamination.

### Virus stocks.

The SFV strain ppSFV (76) and VSV Indiana strain ppVSV (27) are well-characterized plaque-purified isolates and were propagated on BHK-21 cells. SFV, SFV C-M113,115A, SFV Δ6K/TF, and CHIKV 181/25 virus stocks were generated by *in vitro* transcription of viral RNAs and electroporation into BHK-21 cells as previously described (73, 77, 78). Virus-containing culture media were clarified by centrifugation at 7,656 × *g* for 20 min at 4°C and titer was determined by plaque assays on BHK-21 cells.

### Berberine chloride preparation.

Berberine chloride (BBC) was purchased from Sigma-Aldrich (catalog no. B3251) and stored in the dark under desiccating conditions. Fresh stock solutions of BBC were prepared for each experiment. For cell culture experiments, 50 mM BBC stock was prepared in sterile, tissue culture-grade 100% dimethyl sulfoxide (DMSO), incubated in the dark for 30 min at 37°C with intermittent vortexing, and then sonicated at room temperature for two bursts of 2 min each in a bath sonicator (Avanti). For *in vitro* experiments, 2.5 mM BBC was prepared in molecular grade water to avoid potential denaturing effects of DMSO on RNA. The stock was incubated at 37°C for 1 h with intermittent vortexing, filtered through a 0.22-μm syringe filter, and buffered to a final concentration of 2.5 mM BBC in 25 mM K-HEPES (pH 7.4) and 100 mM KCl immediately before addition to *in vitro* reaction mixtures.

### RNA and RNA folding.

Methylated and capped SFV gRNA was prepared as described above. The DNA template was removed by digestion with DNase I (New England Biolabs), and the gRNA was column purified using the RNeasy MinElute kit (Qiagen) and eluted with nuclease-free water (Ambion). RNA purity was assessed by electrophoresis on native 1% (wt/vol) agarose Tris-acetate-EDTA (TAE) (40 mM Tris base [pH 8.3], 20 mM acetic acid, and 1 mM EDTA) gels, and RNA integrity was assessed by electrophoresis on denaturing 1% (wt/vol) agarose MESA-formaldehyde (20 mM MOPS pH 7.0, 1 mM EDTA, 5 mM sodium acetate, and 2.2 M formaldehyde) gels, and detected by SYBR Gold (Invitrogen) staining.

To prepare folded gRNA, purified gRNA was first denatured by heating 25 μl of a <1 μM RNA solution in renaturation buffer (25 mM K-HEPES [pH 7.4], 100 mM KCl, and 1 mM EDTA) at 95°C for 3 min, then renatured by rapid cooling on ice for 1 min to allow formation of stable secondary structures, followed by incubation for 1 h at 37°C in folding buffer (25 mM K-HEPES [pH 7.4], 100 mM KCl, 1 mM EDTA, and 5 mM MgCl_2_) to promote secondary and tertiary structures. RNA samples were aliquoted into nuclease-free, DNA LoBind tubes (Eppendorf), snap-frozen in liquid nitrogen, stored at −80°C, and discarded after thawing. RNA concentrations were estimated by *A*_260_ using an extinction coefficient of 40 ng-cm/μl and a molecular mass of SFV gRNA of 3715717.447 g/mol.

### DNA 48-mer and 48-mer–3′ biotin.

ssDNA 48-mer and 48-mer with a 3′ biotin modification were purchased from Integrated DNA Technologies, resuspended in nuclease-free water to 100 μM, aliquoted, and stored at −20°C. The oligonucleotide sequence is 5′-CCGTTAATGCATGTCGAGATATAAAGCATAAGGGACATGCATTAACGG-3′, as previously described (39, 43).

### Recombinant capsid protein expression and purification.

Expression of 2×Strep-Cp in Escherichia coli Rosetta 2(DE3) (Novagen) was induced by addition of 1 mM isopropyl-β-d-thiogalactopyranoside (IPTG), and cells were grown overnight at 16°C. Cell pellets were disrupted by sonication in buffer W (100 mM Tris-HCl [pH 8.0], 150 mM NaCl, and 1 mM EDTA) with Roche complete protease inhibitor cocktail, DNase I, and 2 mM *N*-ethylmaleimide (NEM). The soluble lysate was then diluted to 10× the volume in high-salt buffer W (100 mM Tris-HCl [pH 8.0], 1.5 M NaCl, and 1 mM EDTA) to dissociate any preformed Cp-nucleic acid complexes, then purified by affinity chromatography using a Strep-Tactin Superflow high-capacity column (IBA) and the manufacturer’s protocol but using high-salt buffer W as the wash buffer. Pooled fractions were dialyzed against TN buffer (50 mM Tris and 100 mM NaCl [pH 7.4]) and concentrated by Amicon Ultra-15 (Millipore). TEV-Strep was expressed in E. coli BL21(DE3) (Novagen) cells, purified by affinity chromatography by Strep-Tactin Superflow, and concentrated in TN buffer. Protein concentrations were determined by *A*_280_.

The affinity tag on 2×Strep-Cp was removed by digestion of 1 mg 2×Strep-Cp with 200 μg TEV-Strep in 1 ml of TN buffer supplemented with 0.5 mM EDTA and 1 mM dithiothreitol (DTT) on ice overnight. The cleaved Cp was purified by passing two times over a Strep-Tactin Superflow high-capacity column, exchanged into 25 mM K-HEPES (pH 7.4) and 100 mM KCl using PD-10 desalting columns (GE Healthcare), and concentrated. Protein purity was confirmed by SDS-PAGE followed by Coomassie staining and Western blotting (WB) analysis using polyclonal antibody (PAb) to the Strep tag (GenScript) and mouse monoclonal antibody (MAb) 2-3 against the Cp (from Irene Greiser-Wilke) (79). The concentration was estimated from *A*_280_ using a molar extinction coefficient for Cp of 37,930 M^−1^ · cm^−1^. Only fractions with an *A*_260_/*A*_280_ ratio of ≤0.6 were used for assembly and binding reactions to ensure that the protein was free of nucleic acid contamination. Cp was aliquoted into Protein LoBind tubes (Eppendorf), snap-frozen in liquid nitrogen, stored at −80°C, and discarded after thawing. The unassembled state of purified Cp was confirmed by sucrose gradient sedimentation, DLS, and negative-stain electron microscopy, as described below.

### Neutral red cytotoxicity test.

The neutral red uptake assay for cell viability was previously described in detail (80) and is adapted here. In brief, 1.5 × 10^4^ BHK-21 cells were seeded into 96-well plates and cultured for 24 h in phenol red-free complete growth medium. Cells were then incubated for 8 h or 24 h at 37°C with serial dilutions of 100% DMSO and 50 mM BBC prepared in phenol red-free medium A (minimal essential medium [MEM], 2 mM glutamine, 0.2% bovine serum albumin [BSA], 100 U/ml penicillin, 100 μg/ml streptomycin, and 10 mM HEPES [pH 7.0], medium S (BHK growth medium with 10 mM HEPES [pH 7.0] and 2% FBS instead of 5%), or 2% FBS medium (medium A with 2% FBS instead of BSA). Cells were then incubated for 2 h with 40 μg/ml neutral red solution in phenol red-free complete growth medium, washed, and extracted, and neutral red absorbance was measured from the top on a SpectraMax M5 microplate reader (Molecular Devices) with SoftMax Pro 7.0 software (Molecular Devices). A dose-response curve with a least-squares (ordinary) fit was drawn using Hill function analysis to determine the concentration of BBC causing 50% inhibition of uptake, which was considered the 50% cytotoxic concentration (CC_50_) of BBC.

### Standard BBC test conditions.

The following are standard conditions, with any minor alterations indicated in individual figure legends. BHK-21 cells (0.4 × 10^6^) were seeded in 6-well plates for 24 h or Vero cells (0.6 × 10^6^) were seeded in 6-well plates for 22 h. Infection with the indicated viruses was performed at an MOI of 10 in medium A for 1 h in BHK-21 cells or for 1.5 h in Vero cells, unless otherwise indicated. Cells were then washed three times and cultured in medium A at 37°C. At 4 h postinfection, cells were washed three times and treated with medium A containing DMSO or BBC, as indicated. Samples were harvested at 8.5 h postinfection and analyzed as described for specific experiments.

To quantitate infectious particle production, culture supernatants were collected at 8.5 h postinfection and clarified by centrifugation at 10,600 × *g* for 5 min at 4°C. Infectious virus was quantitated by plaque assays.

### Steady-state viral protein analysis.

At 8.5 h postinfection, cells were lysed in Triton X-100 lysis buffer (1% vol/vol Triton X-100, 50 mM Tris-HCl [pH 7.4], 100 mM NaCl, 1 mM EDTA, 1 μg/ml pepstatin, and Roche complete protease inhibitor cocktail). Lysates were clarified by centrifugation at 20,800 × g for 10 min at 4°C. 2% of the cleared lysates and 5% of the solubilized pellets were denatured in SDS sample buffer (4% vol/vol SDS, 200 mM Tris base [pH 8.8], 10% glycerol, 0.02% and bromophenol blue) and heated to 95°C for 5 min. The pellet samples were treated with 50 U Benzonase (Sigma-Aldrich) for 30 min at 37°C, followed by nuclease inhibition with 2 mM EDTA. Samples were separated by 11% Tris-glycine SDS-PAGE and analyzed by WB using the following antibodies: anti-SFV E2/E1 rabbit PAb (81), mouse MAb 2-3 against Cp (described above), and anti-histone H3 acetyl K27 rabbit PAb (Abcam). Mouse MAb E7 against β-tubulin, developed by Michael Klymkowsky (University of Colorado), was obtained from the Developmental Studies Hybridoma Bank, created by the NICHD of the NIH and maintained at The University of Iowa, Department of Biology, Iowa City, IA. Detection used Alexa Fluor 680 (AF680)- or DyLight800-conjugated goat anti-mouse or anti-rabbit secondary antibodies (Invitrogen) and the Odyssey Fc imaging system (LI-COR Biosciences) with 685- and 785-nm lasers and Image Studio software 5.2.5 (LI-COR Biosciences).

### Pulse-chase analysis.

At 4.5 h postinfection, cells were starved for 20 min in medium 2 (DMEM without l-methionine or l-cystine and with 25 mM d-glucose, 2 mM l-glutamine, 100 U/ml penicillin, 100 μg/ml streptomycin, and 10 mM HEPES [pH 7.2]) containing DMSO or BBC, pulse-labeled for 30 min with 100 μCi/ml [^35^S]methionine/cysteine in medium 2 supplemented with DMSO or BBC, and then chased in the presence of a 10× excess of methionine/cysteine plus DMSO or BBC. At the indicated chase time points, the cells were lysed, and the samples were immunoprecipitated with an E2/E1 PAb overnight at 4°C. Samples were analyzed by SDS-PAGE and fluorography using a Storm 860 phosphorimager (Molecular Dynamics).

### Specific infectivity determination.

Supernatants were harvested and clarified at 8.5 h postinfection. Titer of an aliquot was determined by plaque assay, and separate aliquots were pelleted through a cushion of 20% (wt/wt) sucrose in TN buffer by centrifugation in a SW41 Ti rotor at 35,000 rpm for 3 h at 4°C. Virus pellets were resuspended on ice overnight in TNE buffer (50 mM Tris-HCl [pH 7.4], 100 mM NaCl, 1 mM EDTA, and Roche complete protease inhibitor) plus 1,000 U/ml RNase inhibitor for RNA isolation.

To determine PFU:particle ratio, resuspended virus samples were analyzed by SDS-PAGE and WB using saturating levels of an E2-specific MAb E2-1 (82), followed by detection with AF680-conjugated goat anti-mouse antibody and the Odyssey Fc imaging system.

To determine PFU:genome ratio, RNA was isolated from resuspended virus samples using the MagMax viral RNA isolation kit (Applied Biosystems). First-strand cDNA was synthesized from 10% RNA input using the Verso cDNA synthesis kit (Thermo Scientific) and 50 nM SFV sequence-specific reverse primer (nsP3, 5′-GGCTATGTCTGCTCTCTTAACTC-3′). Real-time qPCR was then performed on the Applied Biosystems ViiA 7 real-time PCR system and software using 20% of the cDNA input, Power SYBR green PCR mastermix (Applied Biosystems), and 200 nM (each) SFV sequence-specific forward (nsP2, 5′-CTACGCTACACCAGATGAATACC-3′) and reverse primers in a total volume of 10 μl in a 384-well clear PCR plate (Axygen) sealed with adhesive sealing film (Bio-Rad). Cycling parameters were 50°C for 2 min and 95°C for 10 min and 45 cycles of 95°C for 15 s and 60°C for 60 s, followed by melt curve analysis. The number of gRNA copies was determined by relating the quantification cycle values (*C_q_*) to genome copies via a standard curve generated from 10-fold dilutions of 0.5 ng of *in vitro*-transcribed SFV gRNA (8.1 × 10^7^ to 8.1 copies). *In vitro*-transcribed SFV gRNA was prepared as described above, and then DNA templates were digested with Turbo DNase I (Ambion) for 30 min at 37°C, and RNA was purified using an RNeasy MinElute kit. RT-qPCRs without reverse transcriptase were performed to ensure ≤0.05% residual DNA template contamination. Mock-infected, treated cell supernatants, as well as no-template controls, were used to ensure viral RNA-specific amplification. Paired statistical analyses were assessed on six independent sample biological replicates, four standard curve replicates, and three technical repeats.

### Transmission electron microscopy.

BHK-21 cells (0.3 × 10^6^) were seeded in 35-mm dishes for 24 h and infected with ppSFV and BBC treated using the standard conditions. Culture supernatants were collected at 8.5 h to confirm titer differences between vehicle and treated samples. Cell monolayers were washed once with warm serum-free growth medium and fixed with 2.5% glutaraldehyde in 0.1 M sodium cacodylate buffer for 1 h at room temperature. The Einstein Analytical Imaging Facility processed, embedded, and sectioned the samples and stained the sections with uranyl acetate followed by lead citrate. Samples were viewed in a blind manner on a JEOL 1200EX transmission electron microscope operating at 80 kV and equipped with a Gatan Orius Digital 2k × 2k charge-coupled device (CCD) camera.

### Immunofluorescence analysis.

Vero cells (1.5 × 10^4^) were seeded in Nunc Lab-Tek II 8-well no. 1.5 glass chambers (Thermo Scientific) for 22 h, infected for 2 h at 37°C with ppSFV at an MOI of 10, cultured, and treated with DMSO or BBC per the standard protocol. At 8.5 h postinfection, cells were fixed with fresh 4% paraformaldehyde in phosphate-buffered saline and processed for immunostaining with or without permeabilization, as indicated in the legends, and using the following antibodies: mouse MAb E1-1 against E1 (82), anti-SFV E2/E1 rabbit PAb, mouse MAb 2-3 against Cp, mouse MAb E2-1 against E2, mouse MAb E7 against β-tubulin, and isotype and species-specific goat secondary antibodies conjugated to AF405, AF488, or AF568 (Invitrogen). Nuclei were stained with Hoechst 33342. Cells were imaged on a Leica SP8 confocal microscope with LAS X software, a 63× oil objective (numerical aperture 1.4), and HyD hybrid detectors using the same settings for DMSO and BBC samples, and image processing was performed using ImageJ v. 1.51u.

### Analysis of cytoplasmic nucleocapsids in infected cells.

At 4.5 h postinfection, cells were labeled with 50 μCi/ml [^35^S]methionine/cysteine in medium 2 supplemented with DMSO or BBC for 4 h at 37°C. Cells were then lysed in NP-40 lysis buffer (1% vol/vol NP-40, 100 mM Tris-HCl [pH 7.4], 50 mM NaCl, 2 mM EDTA, 10 mM iodoacetamide, 250 U/ml RNase inhibitor, 1 μg/ml pepstatin, and Roche complete protease inhibitor cocktail). After centrifugation at 3,800 × g for 5 min at 4°C, NP-40 insoluble pellets were frozen at −80°C, and lysates were incubated with 25 mM EDTA on ice for 20 min to dissociate polysomes. Lysates were centrifuged on 7.5 to 20% (wt/wt) sucrose gradients in TN buffer, 2 mM EDTA, 0.1% vol/vol NP-40, with a 60% (wt/wt) sucrose cushion without NP-40 using the SW41 Ti rotor at 41,000 rpm for 2 h at 4°C. Fractions (0.5 ml) were collected, and the bottom of the tube was washed out with SDS sample buffer. The NP-40 insoluble pellets were thawed and resuspended in SDS lysis buffer (1% [vol/vol] SDS, 50 mM Tris-HCl [pH 7.4], 100 mM NaCl, 1 mM EDTA, 10 mM iodoacetamide, 1 μg/ml pepstatin, and Roche complete protease inhibitor cocktail) and treated with 50 U Benzonase as above. Aliquots of the samples were analyzed by SDS-PAGE and fluorography using a FLA-5100 fluorescent image analyzer (Fujifilm) at 635 nm and ImageReader FLA-5000 series v. 1.0 application. Image processing was performed using MultiGauge analysis software (Fujifilm). Aliquots of the fractions and lysates were also quantitated by scintillation counting.

### Analysis of VLP-expressing cells.

BHK-21 cells (0.3 × 10^6^) were seeded in 6-well plates for 24 h and transfected with 4 μg/well of pcDNA3.1-SFV Cp626KE1 in Opti-MEM using Lipofectamine 2000. At 5 h posttransfection, the transfection medium was replaced with complete growth medium for 1 h, then replaced with BHK medium S with 10 mM HEPES [pH 8.0]. At 22 h posttransfection, cells were washed three times with medium A, then treated with medium A supplemented with DMSO or BBC for 8 h. At 30 h posttransfection, culture media were collected, and the cells were lysed and processed as above. Clarified lysates were loaded onto 10 to 30% (vol/vol) iodixanol gradients (OptiPrep; Sigma-Aldrich) in TN buffer (pH 7.4) with 2 mM EDTA and 0.1% (vol/vol) NP-40 overlaid on a 50% (vol/vol) iodixanol cushion without NP-40 and centrifuged and fractionated as above. VLP-containing media were clarified by centrifugation at 10,600 × g for 5 min at 4°C, pooled together from 3 wells, layered over a 20% (wt/wt) sucrose cushion in TN buffer (pH 7.4) and pelleted in an SW41 Ti rotor at 35,000 rpm for 3 h at 4°C. VLP pellets, cell lysates, and gradient fractions were separated by SDS-PAGE and analyzed by WB using the primary antibodies described in Fig. 5. Transfection with pcDNA3.1-SFV p626KE1 served as a control to show that Cp was required for detectable VLP formation.

### Core-like particle assembly reactions.

*In vitro* CLP assembly assays have been previously described using recombinant alphavirus Cp and gRNA, ssDNA, or ssRNA as the nucleic acid (38–44). DNA assembly buffer (25 mM K-HEPES [pH 7.4], 100 mM KCl, and 2 mM MgCl_2_), gRNA assembly buffer (25 mM K-HEPES [pH 7.4] and 100 mM KCl), and folded gRNA assembly buffer (25 mM K-HEPES [pH 7.4], 100 mM KCl, and 5 mM MgCl_2_) were freshly made just before use. Concentrated stocks of nucleic acids and Cp were thawed on ice, diluted separately into assembly buffer, then equilibrated for 30 min at room temperature. Aliquots (20 μl) of assembly buffer containing final concentrations of 2.5 μM Cp plus 1.25 μM DNA (2:1 Cp:DNA molar ratio), 10 nM RNA or folded RNA (250:1 Cp:RNA molar ratio), or 3.47 nM RNA or folded RNA (720:1 Cp:RNA molar ratio) were incubated for 30 min (DNA) or 1.5 h (RNA) at 22°C. For assembly reactions containing BBC, Cp and the nucleic acids were first diluted separately into assembly buffer and equilibrated for 30 min, and then BBC was incubated with nucleic acids for 5 to 10 min at 22°C before Cp was added. All experiments were reproduced using different protein and RNA preparations.

### Dynamic light scattering data collection and analysis.

DLS measurements were performed on a DynaPro Plate Reader (Wyatt Technology) and acquired, analyzed, and visualized using DYNAMICS v. 7.8 software (Wyatt Technology). Light scattering was achieved by an 830-nm laser, which is outside the excitation and emission wavelengths for BBC. Power was set to autoattenuation to automatically adjust for optimal signal-to-noise ratios in real time, and normalized intensities (count/s) were collected. Assembly reactions of 20 μl were read at 22°C in a low-volume, nontreated 384-well black plate with a clear flat polystyrene bottom (Corning) after a 1-min spin at 2,000 rpm to remove bubbles. Plates were covered with sealing film (Axygen) whenever possible to minimize dust introduction and sample evaporation, but film was removed for data acquisition. Measurements consisted of 10 read acquisitions of 10 s, and measurements were averaged and displayed as intensity ACFs. Truncation lines for ACF fitting were set at 1.5 μs and 2.5 × 10^6^ μs. Outliers, contaminants, or other sources of noise with poor ACFs were first filtered with baseline limits set at 0.01, minimum amplitude set at 0.05, maximum amplitude set at 1, and maximum sum of squares set at 100. Measurements were then checked for any remaining invalid ACFs (defined as those lacking a smooth and continuous curve and exponential decay), which were marked and removed from fit calculations. The Dynals regularization algorithm was applied to the ACF and checked for fitness, and the *R_h_*, % Pd, and relative abundance of all species present in solution were determined. The peak radii cutoffs were set at 0.50 nm and 10 μm. To facilitate comparisons, ACFs were adjusted so their amplitudes were displayed at the same relative value.

### Negative-stain transmission electron microscopy.

Carbon-coated 400-mesh copper grids were rendered hydrophilic for sample binding by glow discharging for 2 min at 46 V under 150 mtorr in a Denton DV-502 vacuum evaporator. Samples (5 μl) were applied to freshly glow-discharged grids, stained with 1% (vol/vol) uranyl acetate solution, and air dried, then viewed on a FEI Tecnai 20 transmission electron microscope operating at 120 kV and equipped with a TVIPS F-415 4k × 4k CCD camera.

### Binding assay by competitive ELISA.

Nontreated medium-binding 96-well plates (Corning) were incubated with 100 ng Cp/well in coating buffer (0.1 M Na_2_CO_3_ and 0.1 M NaHCO_3_ [pH 9.6]) for 3 h at 37°C. Stock solutions of 48-mer**–**3′ biotin were diluted to 334.2 nM and incubated for 5 to 10 min at room temperature with serial dilutions of BBC starting at a 400:1 BBC:DNA molar ratio in DNA assembly buffer. Plates were washed, and the BBC-DNA reaction mixtures, representing a 10× molar excess of 48-mer over Cp, were allowed to bind to Cp for 1 h at 37°C under gentle agitation (300 rpm). Plates were washed, then blocked with 1% BSA, and the bound biotin was probed with streptavidin conjugated to HRP (Thermo Fisher Scientific) for 1 h at room temperature. After washing, streptavidin was detected using 100 μl 1-Step Ultra-TMB substrate (Thermo Scientific) for 15 min at room temperature under vigorous shaking (800 rpm). The reaction was stopped by adding 100 μl of 2N H_2_SO_4_, and the *A*_450_ was measured from the top on the SpectraMax M5 microplate reader with SoftMax Pro 7.0 software.

### Statistical analysis.

Where stated, analyses were performed using Prism 8.3.1 for MacOS (GraphPad Software). One-way or two-way analysis of variance (ANOVA) followed by Dunnett’s or Bonferroni’s multiple-comparison test was performed to assess statistical significance between unpaired sample means compared to a control mean or a set of means with a Gaussian distribution. Two-way repeated-measures ANOVA with Bonferroni’s multiple-comparison test was performed to assess statistical significance between paired sample means comparing WT versus mutant virus. Paired or multiple *t* tests were performed to assess statistical significance of unpaired sample means with a Gaussian distribution. The Wilcoxon signed-rank test was performed to assess the statistical significance of paired sample means between vehicle versus BBC treatment. Data in all graphs represent the means from two to seven independent experiments, with individual data points plotted in lieu of error bars (83).
