# Supplementary figures and images for: Berberine Chloride is an Alphavirus Inhibitor That Targets Nucleocapsid Assembly
Source: mBio. 2020 Jun 30;11(3):e01382-20. doi: 10.1128/mBio.01382-20 (PMC7327175; doi:10.1128/mBio.01382-20)

A

E2-AF568

E1-AF488

Nuclei (Hoescht)

Composite

0.1% DMSO

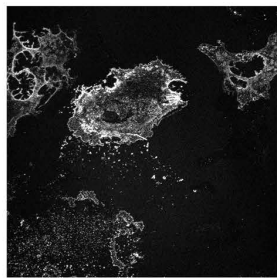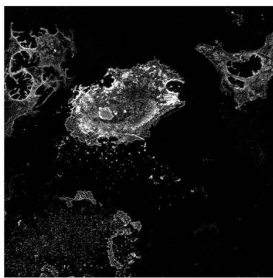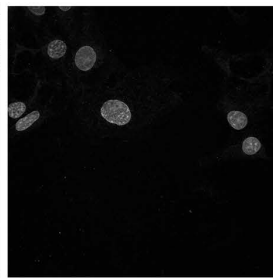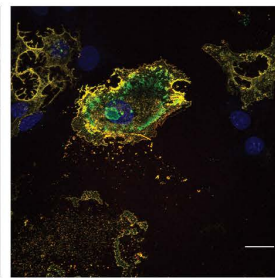50  $\mu$ M BBC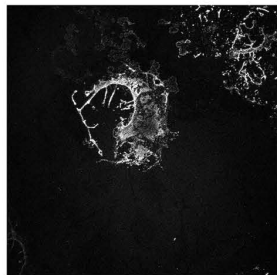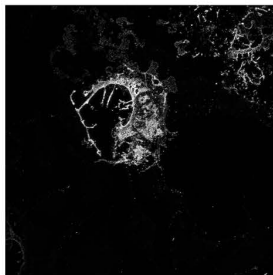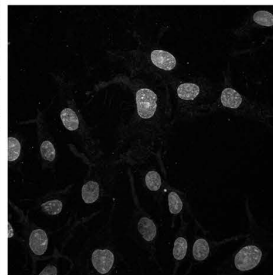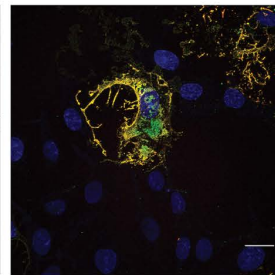

B

E2/E1-AF405

Cp-AF568

Tubulin-AF488

Composite

0.1% DMSO

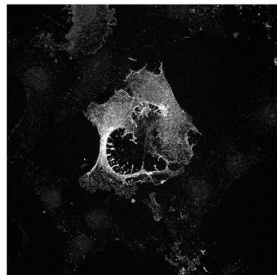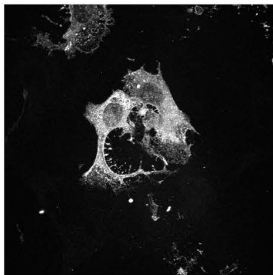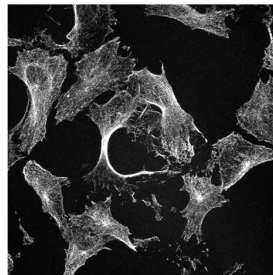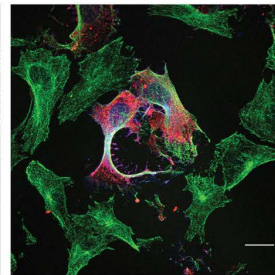50  $\mu$ M BBC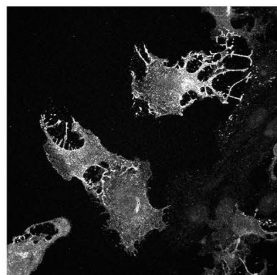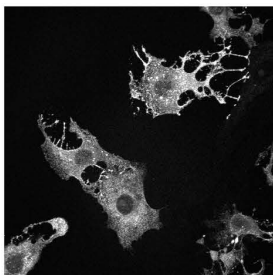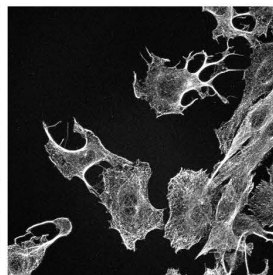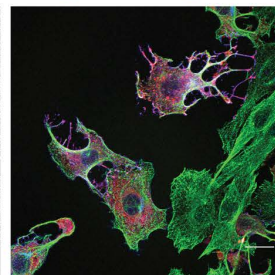

Supplement: FIG S3 [file mBio.01382-20-sf003.pdf]

**A****DMSO**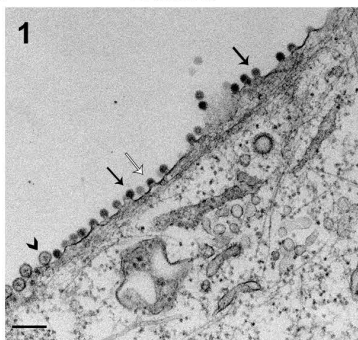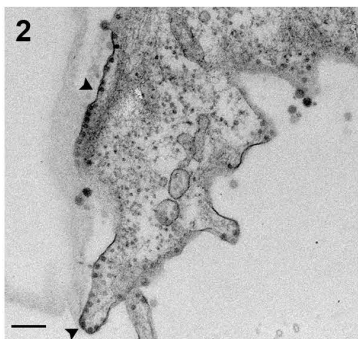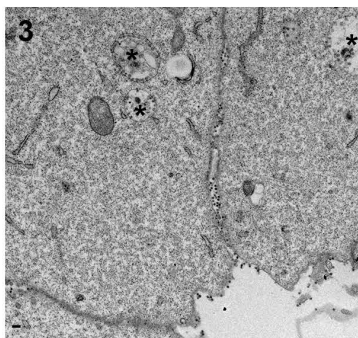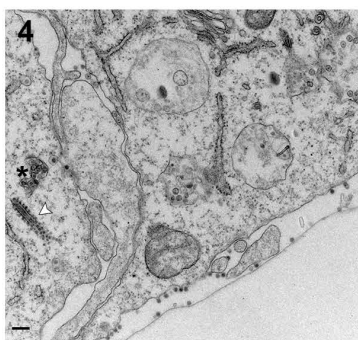**B****BBC**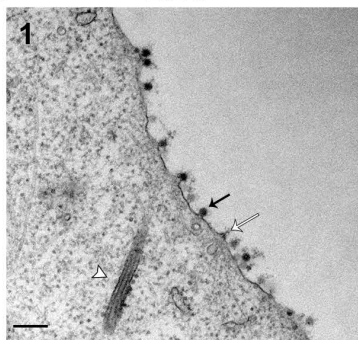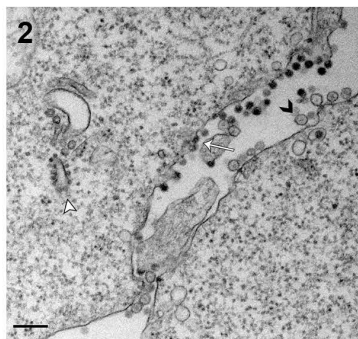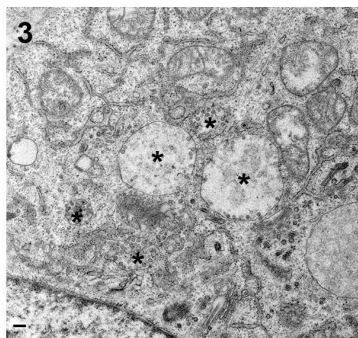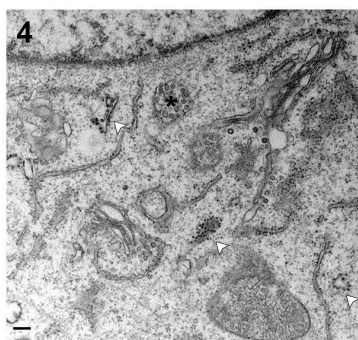

Supplement: FIG S4 [file mBio.01382-20-sf004.pdf]

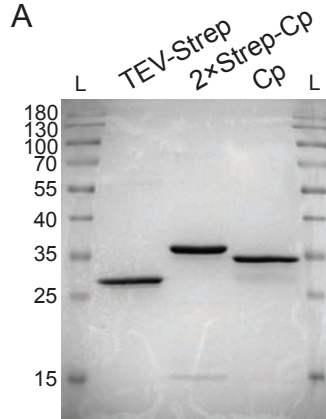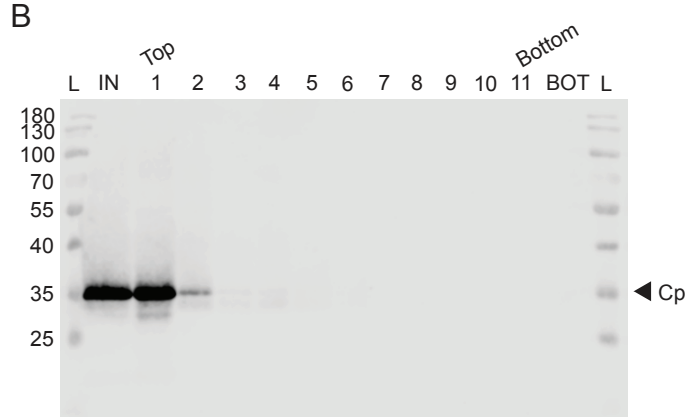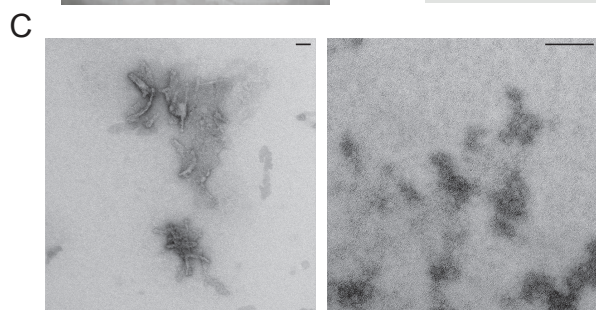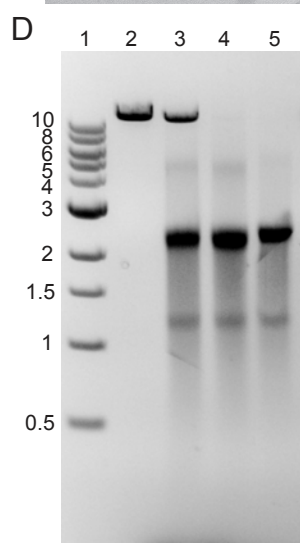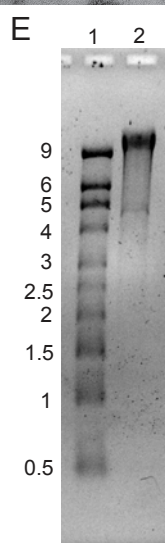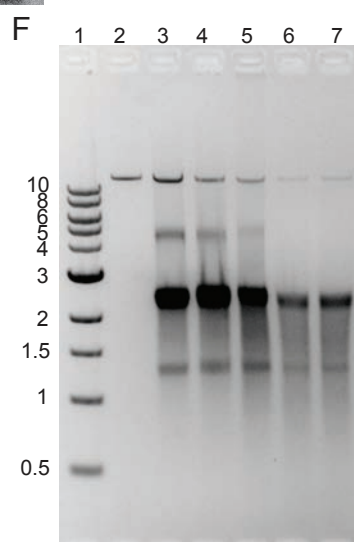

Supplement: FIG S5 [file mBio.01382-20-sf005.pdf]

**A**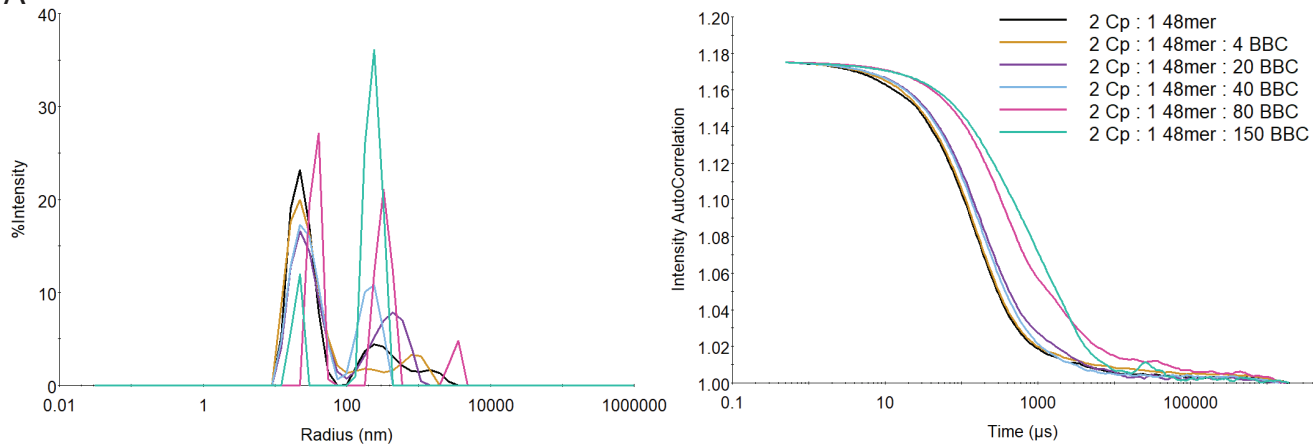**B**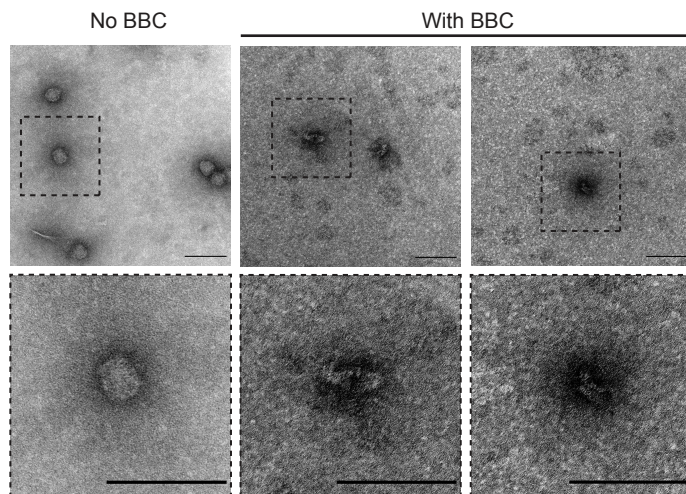

Supplement: FIG S8 [file mBio.01382-20-sf008.pdf]
